# Persistent pain and numbness in the extremities of an adult due to paraneoplastic peripheral neuropathy caused by olfactory neuroblastoma: A case report

**DOI:** 10.3389/fneur.2022.1002076

**Published:** 2023-01-09

**Authors:** Wenwen Liang, Yanyan Wang, Wenzhe Sun, Dongrui Li, Xiaoping Zhang, Pengcheng Zhu, Zhou Zhu, Yongkang Fang

**Affiliations:** ^1^Department of Neurology, Tongji Medical College, Tongji Hospital, Huazhong University of Science and Technology, Wuhan, Hubei, China; ^2^Department of Neurology, The First Affiliated Hospital of Zhengzhou University, Zhengzhou, Henan, China; ^3^Department of Infection, Tongji Medical College, Tongji Hospital, Huazhong University of Science and Technology, Wuhan, Hubei, China; ^4^Department of Pathology, Tongji Medical College, Tongji Hospital, Huazhong University of Science and Technology, Wuhan, Hubei, China

**Keywords:** paraneoplastic peripheral neuropathy, olfactory neuroblastoma, anti-Hu antibody, case report, FDG-PET/CT

## Abstract

**Background:**

Paraneoplastic peripheral neuropathy (PPN) caused by olfactory neuroblastoma (ONB) has not yet been reported.

**Case report:**

We present a rare case of an adult who hospitalized repeatedly over the past 9 months for persistent pain and numbness in the limbs. This patient was initially diagnosed with chronic inflammatory demyelinating polyneuropathy (CIDP) and treated accordingly, but neurological symptoms did not improve significantly. After this admission, FDG-PET/CT showed focal hypermetabolism of a soft-tissue mass in the nasal cavity, and further lesion biopsy suggested ONB. Combined with positive serum anti-Hu antibody, the diagnosis of PPN associated with ONB was eventually made. Furthermore, the patient's neurological symptoms were relieved after removal of the primary tumor, confirming the accuracy of the diagnosis.

**Conclusion:**

Our case not only expanded the clinical characteristics of ONB but also highlighted the importance of early and comprehensive tumor screening for the diagnosis of PPN.

## Introduction

Paraneoplastic peripheral neuropathy (PPN) has a variety of clinical manifestations, with numbness, pain or paresthesia in one or multiple limbs being the most common. Some patients can also present with weakened muscle strength, weakened or disappeared tendon reflexes, and muscular atrophy. A few individuals even show sensory ataxia and postural tremor (1). However, the pathogenesis of PPN has not been fully elucidated, and immune response triggered by the tumor is considered as the most likely cause (2). Based on a host of previous studies, we know that PPN is commonly associated with small-cell lung cancer (SCLC) (3) and lymphoma (4), but has not been reported in olfactory neuroblastoma (ONB).

ONB is a rare nasal malignant neoplasm that has been proven to originate in the neuroectoderm (5). There is no significant difference between male and female incidence. The age of onset is bimodal, and the peak of onset is 10–20 and 50–60 years (6). Patients with ONB most commonly present with unilateral nasal obstruction (70%) followed by epistaxis (41%), which are fairly non-specific (7). A few patients may have seizures, pituitary dysfunction and other manifestations (8).

In this study, we report for the first time such a rare case-an adult patient with persistent pain and numbness in the limbs was eventually diagnosed with PPN caused by ONB. This unique case highlights a rare cause of PPN and the uncommon clinical presentation of ONB.

## Case

A patient was admitted to the Department of Neurology of our hospital with persistent numbness and pain in limbs for 9 months. This adult had a history of rhinitis and a weight loss of 5 Kg over the past 3 months. Prior to this, the patient had repeatedly visited local hospitals for treatment, but the symptoms were not relieved. Now, the diagnosis and treatment process of these several hospitalizations were presented in chronological order as follows.

On July, 2021 the patient felt numbness and pain in the extremities without obvious inducement, which was not taken seriously because the symptoms were mild. Two months later, the pain and numbness of the extremities became worse than before, and the obvious symmetrical continuous needle-like pain made him unbearable. Therefore, he was firstly hospitalized in a local hospital on September 23, 2021. Cerebrospinal fluid (CSF) results revealed a phenomenon of protein cell separation -total protein concentration increased dramatically (3,273 mg/L) while cell counts remained normal (2 × 10^6^/L). Electromyography (EMG) showed neurogenic damage around both upper limbs involved the myelin sheath of the sensory axon-the motor nerve conduction velocity (MCV) of bilateral median nerve and ulnar nerve was normal, but the sensory nerve conduction velocity (SCV) was slow (left median nerve:38.4 m/s, right ulnar nerve:35.7 m/s). The compound muscle action potential (CMAP) amplitude of the right median nerve and ulnar nerve was normal, but the sensory nerve action potential (SNAP) amplitude was reduced (right median nerve:7.22 μV, right ulnar nerve:7.20 μV). The latency and occurrence rate of F wave in bilateral median nerve were normal. Based on the insidious onset and the chronic progression of the disease, the phenomenon of protein cell separation, and the results of the EMG, the individual was initially diagnosed as peripheral sensory neuropathy: pure sensory chronic inflammatory demyelinating polyneuropathy (CIDP). After treatment with glucocorticoids and neuroprotective drugs, the numbness and pain of the limbs did not improve significantly.

On January 1, 2022, the patient was admitted to another local hospital for a sudden loss of awareness and limb convulsions as well as the persistent numbness and pain in limbs. CSF results indicated a significantly elevated total protein level (1330 mg/L) and slightly elevated cell count (10 × 10^6^/L). However, no antiganglioside (GM1 and GQ1b), autoimmune encephalitis (NMDAR and GABABR) and demyelinating antibodies (AQP4, MOG and GFAP) in serum and CSF were detectable. The identification of microorganisms using next-generation CSF sequencing were negative. EMG showed extensively reduced conduction velocities of motor and sensory nerve involving both upper and lower extremities. Electroencephalogram (EEG) showed epileptiform discharges originating from bilateral frontal lobes. Magnetic resonance imaging (MRI) scanning including contrast-enhanced and diffusion weighted imaging (DWI) demonstrated abnormal signals in subcortical areas of the right frontal lobe. The patient was diagnosed with focal epilepsy and peripheral neuropathy. After a series of treatments, including glucocorticoid impulse therapy, intravenous immunoglobulin (IVIG), anti-seizure medication, neuroprotective and anti-infective treatment, the epileptic seizure was controlled while the persistent numbness and pain of limbs had not been significantly ameliorated.

**Figure 1:**
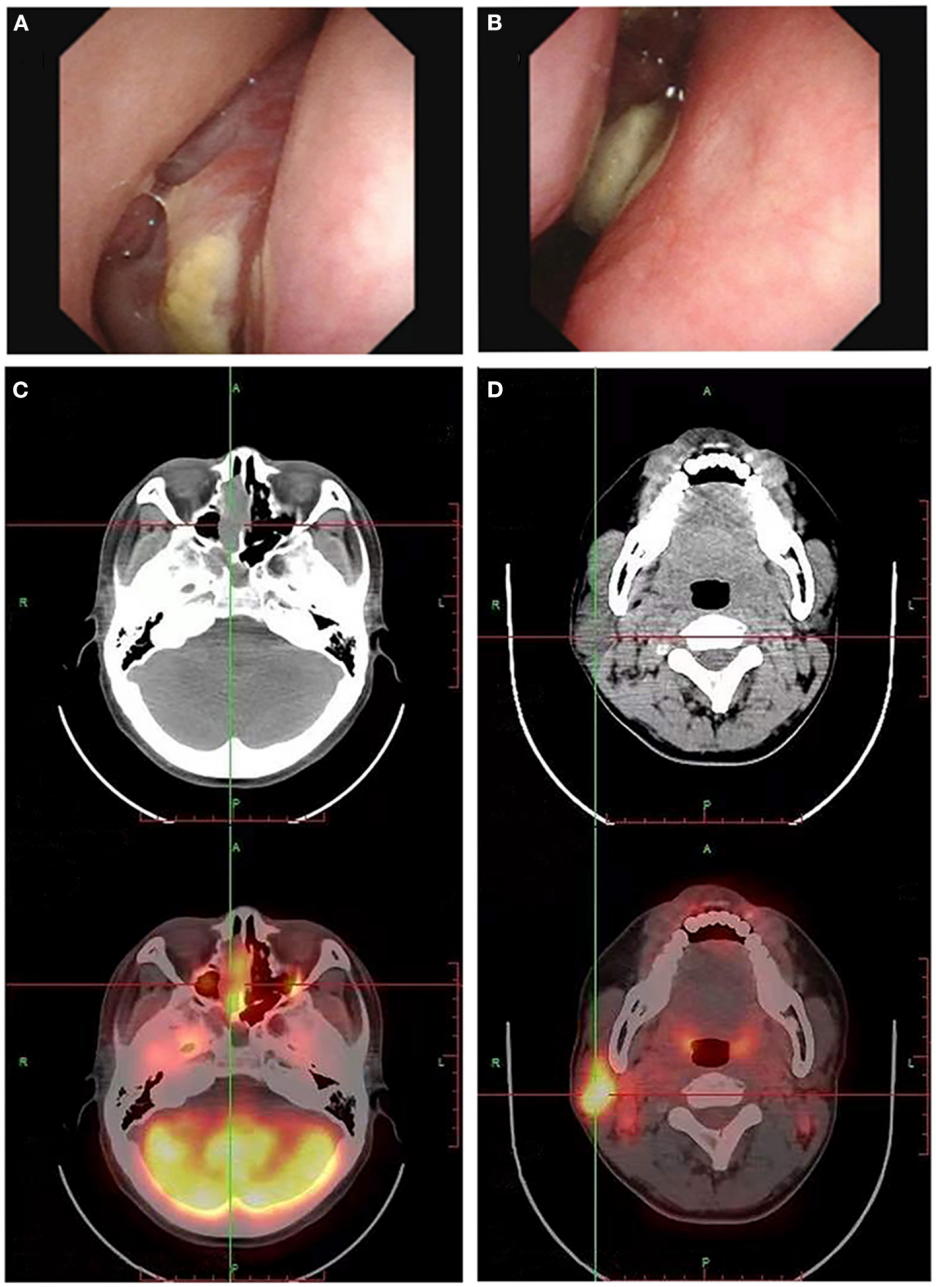
Electronic nasopharyngoscopy reveals a neoplasm in the right nasal cavity **(A, B)**. The fluorodeoxyglucose PET/CT scan may show multiple high metabolic signals in the nasal cavity **(C)** and cervical lymph nodes **(D)**, suggesting the nasal cavity neoplasm growth and lymph node metastasis.

**Figure 2:**
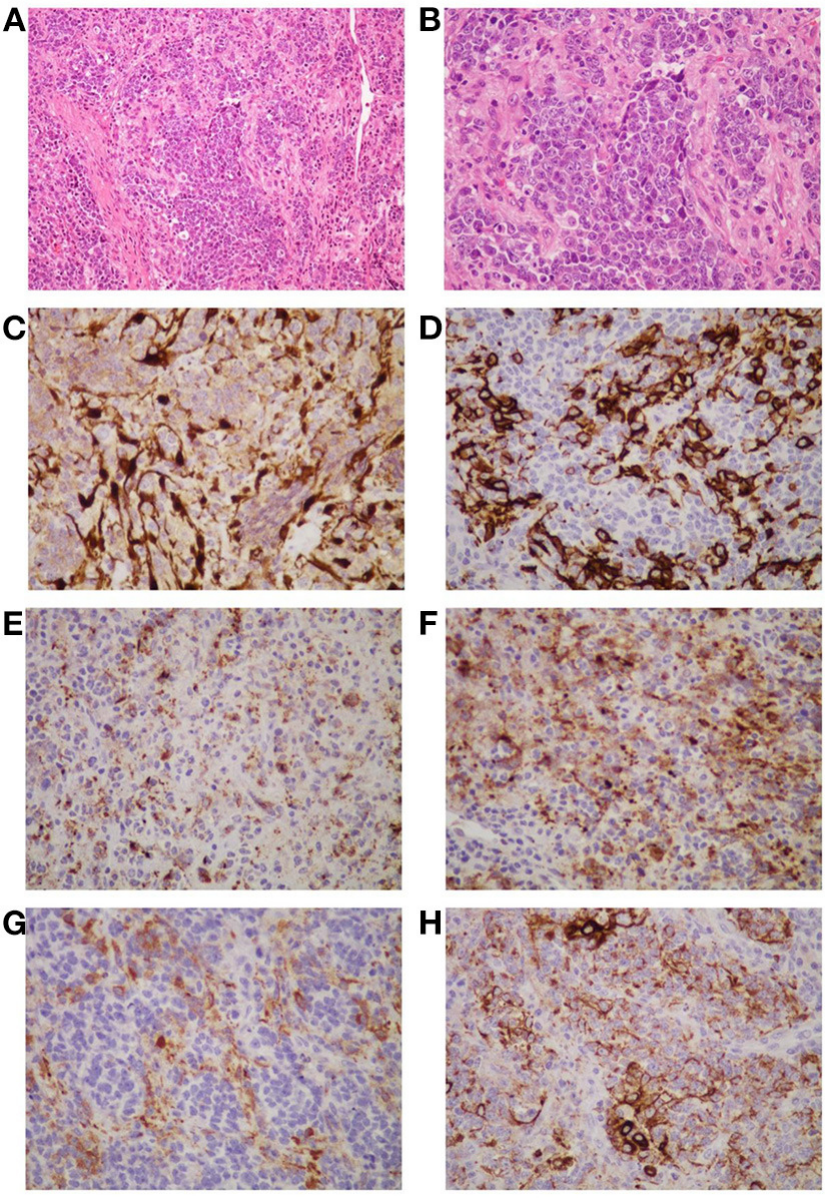
The histopathological appearance of this neoplasm suggests a high-grade round cell malignant tumor, which is consistent with ONB (Hyams grade: IV). The tumor is composed of uniform small round cells that form irregular clusters or islands against a background of hyperplastic vascular and fibrous stroma. The nuclei of the small cells are round or oval, dark stained, uniform in size, and have high mitotic activity. The cytoplasm is stained pink [**(A, B)**, H and E staining]. Immunohistochemistry shows tumor cells are positive for S-100 **(C)**, CD56 **(D)**, chromogranin **(E)**, synaptophysin **(F)**, neuron-specific enolase **(G)**, and pan-cytokeratin **(H)**. The S-100 protein positive cells are located at the periphery of the tumor lobule **(C)**. CD56 gives strong and obvious membrane-type staining in some tumor cells **(D)**. Chromogranin yields a different granular reaction in the cytoplasm of the neoplastic cells **(E)**. Synaptophysin immunoreactivity is positive in partial tumor cells **(F)**. Part of the tumor cells are positive for neuron-specific enolase in the cytoplasm **(G)**. Pan-cytokeratin staining shows dot-like strongly positive expression **(H)**. **(A)** Original magnification × 200; **(B–G)** original magnification × 400.

On April 9, 2022, the patient was hospitalized again due to increased pain and numbness in extremities. After this admission, a detailed physical examination indicated palpable neck lymph nodes, symmetrical tetraparesis, reduction in reflexes, and hyperalgesia. Further fine-needle aspiration of the nodes for cytological examination suggested the presence of metastatic tumor cells, although their origin cannot be unequivocally stated. In addition, FDG-PET/CT showed focal hypermetabolism of a soft-tissue mass in the nasal cavity, suggesting a malignant tumor lesion (Figures 1C, D). To further clarify the association between the patient's peripheral neuropathy and this “nasal tumor” found by accident, the detection of paraneoplastic neuronal antibody in serum was performed. Surprisingly, the results showed that the patient's serum anti-Hu antibody was positive. Eventually, a definitive diagnosis of PPN was made. The patient was subsequently transferred to the department of otolaryngology for resection and biopsy of the nasal mass. Unexpectedly, the pathological results of this neoplasm shown high-grade round cell malignant tumor, which was consistent with ONB. According to the pathological grading standard of Hyams, ONB could be divided into four grades (Table 1). HE staining of this patient presented obvious mitotic figures, significant nuclear pleomorphism, and lack of typical lobular structure, meeting the diagnostic criteria of Hyams grade IV (Figures 2A, B). Meanwhile, immunohistochemistry of neuroblastoma elements showed cells were positive for S-100, CD56, chromogranin, synaptophysin, neuron-specific enolase, and pan-cytokeratin (Figures 2C–H). CD99, P63, FLI1, LCA, HMB45, Desmin and vimentin were negative, supporting the diagnosis of ONB. Proliferation marker studies using Ki-67 revealed a high proliferative index of 40%. After tumor resection and neck lymph nodes dissection, the patient recovered well with nasal ventilation improved. Although the symptoms of numbness and pain in the extremities were still present, they were significantly relieved. The patient is currently undergoing chemotherapy in the Oncology department and will be followed up continuously.

**Table 1:** Olfactory neuroblastoma grading (based on Hyams' grading system) (9).

**Microscopic features**	**Grade I**	**Grade II**	**Grade III**	**Grade IV**
Architecture	Lobular	Lobular	±Lobular	±Lobular
Pleomorphism	Absent to slight	Present	Prominent	Marked
NF matrix	Prominent	Present	May be present	Present
Rosettes	HR	HR	FW	FW
Mitoses	Absent	Present	Prominent	Marked
Necrosis	Absent	Absent	Present	Prominent
Glands	May be present	May be present	May be present	May be present
Calcification	Variable	Variable	Absent	Absent

NF, neurofibrillary; HR, Homer Wright pseudorosettes; FW, Flexner-Wintersteiner rosettes.

## Discussion

Paraneoplastic neurological syndromes (PNS) are a group of nervous system damages caused by distant effects of tumors and their pathogenesis is considered as the action of tumor-derived antibodies or cell-mediated immune response on the nervous system (10, 11). The disorders usually have a subacute onset and cause severe neurological disability (12). PPN is a special type of PNS which mainly involves the peripheral nerve. The application of EMG plays a particularly important role in the diagnosis of PPN. It is traditionally believed that the most classical presentation of PPN is subacute sensory neuropathy (SSN) (13). Whereas, some scholars analyzed the electrophysiological results of PPN patients and found that sensory-motor neuropathy is more commonly seen than simple sensory neuropathy (14). Due to the low incidence and insufficient knowledge of PPN, clinicians' awareness of this disease remains inadequate. Besides, symptoms of peripheral nerve damage usually appear before the primary tumors are diagnosed (15), making a definitive diagnosis of PPN extremely difficult. In previous studies, anti-Hu antibody (also known as type 1 anti-neuronal nuclear antibody) has been confirmed to be the most common autoantibody marker of PPN (16). Anti-Hu antibody is strongly related to lung cancer, mostly SCLC (13, 17). More rarely, it develops in association with extra-thoracic neoplasms such as neuroblastoma or intestinal, prostate, breast, bladder, and ovary carcinomas (18). It has also been proved that patients with positive anti-Hu antibodies can manifest various neurological disorders, including sensory neuropathy, cerebellar ataxia (19), limbic encephalitis (20), brainstem encephalitis (21), myelitis, or intestinal pseudo-obstruction (22).

For this patient, extensive workup was conducted to rule out most potential etiological causes such as diabetic, alcoholic, infectious, autoimmune, and hereditary neuropathies. Combined with the discovery of ONB and positive serum anti-Hu antibody, the patient was finally diagnosed as PPN. It is worth noting that some PPN patients accompanied with motor neuron damage, often have typical demyelination manifestations of EMG, and sometimes protein cell separation can be seen in CSF examination, which may lead to misdiagnosis as acute or chronic inflammatory demyelinating polyneuropathy (AIDP/CIDP) clinically. At present, there is no specific treatment for PPN. Early treatment of primary tumor can alleviate peripheral neuropathy symptoms to a certain extent and prolong the survival time of patients (23). Therefore, it is of great necessity to conduct early screening of the primary tumor as well as detection of paraneoplastic antibodies for patients with peripheral neuropathy.

The seizure occurred in this patient was also thought to be related to ONB. Paraneoplastic limbic encephalitis (PLE), as another extremely rare paraneoplastic syndrome, is usually characterized by epileptic seizures, progressive amnesia, psychological and behavioral abnormalities (24). Previous literature has reported that PLE most commonly involves the limbic systems such as hippocampus and amygdala in the medial temporal lobe (24). The patient had no history of epilepsy and no infection or other pathogenic factors before the onset of that seizure, but head MRI and EEG suggested frontal lobe lesions. Although the lesions were atypical, the epileptic symptoms, positive serum anti-Hu antibodies, and the presence of ONB all supported the diagnosis of paraneoplastic encephalitis.

ONB is a rare malignant tumor of nasal sinuses with an incidence of only 0.4/million, accounting for 3–5% of nasal cavity tumors (25, 26). The pathology of ONB is characterized by a high tendency to invade adjacent organs and tissues, and distant metastasis mainly occurs through lymph nodes and blood (27). Cervical metastasis is the most common in advanced ONB, and cervical lymph node metastasis can occur in about 5–8% of patients (28). ONB can be diagnosed mainly by biopsy or pathological examination after complete resection of the tumor. However, ONB is rich in blood supply and prone to bleeding, so the biopsy generally needs to be performed in the operating room to facilitate hemostasis (29). Modified Kadish stage and Hymans pathological grade are widely used in clinical practice, both of which can provide important guidance for prognosis and treatment of ONB (30). Combined therapy is a widely accepted treatment scheme for ONB at present (31). In terms of improving survival rate and reducing local recurrence rate, surgical resection combined with postoperative radiotherapy usually achieves better results (32, 33). Furthermore, some studies have reported that neoadjuvant chemotherapy can prolong the survival time of locally advanced cases (34).

The clinical manifestations and imaging features of ONB are non-specific, which may greatly increase the difficulty of diagnosis. In clinical practice, attention should be paid to the differential diagnosis of ONB from other small round cell malignant tumors of the nasal cavity, such as squamous cell carcinoma, extra-nodal NK/T cell lymphoma, nasal type, rhabdomyosarcoma, sinonasal undifferentiated carcinoma, neuroendocrine carcinoma, malignant melanoma, Ewing's sarcoma (9). In this case, immunohistochemical analysis of the patient's neuroblastoma elements showed positivity for cells S-100, CD56, chromogranin, synaptophysin, neuron-specific enolase, and pan-cytokeratin, supporting the diagnosis of ONB (9, 35). The negative expression of CD99, P63, FLI1, LCA, HMB45, Desmin and vimentin reduced the possibility of diagnosis of other small round cell malignant tumors of the nasal cavity. Previous studies have shown that ONB usually does not express cytokeratin or shows focal or diffuse expression. However, this patient's immunohistochemical staining showed dot-like cytokeratin expression (Figure 2H). Generally, this expression is a typically important pathologic feature of neuroendocrine cancers (NEC). After an extensive review of the literature, we found that some ONBs can indeed express the dot-like cytokeratin (35, 36). Thus, there is certain difficulty in correctly differentiating high-grade ONB from high-grade NEC, especially in small biopsies. Pathologists should be aware of this potential diagnostic pitfall. Furthermore, ONB can be easily misdiagnosed as nasal polyps as shown in our case who had a history of rhinitis with mild nasal congestion and runny nose as the main clinical manifestations. Three months ago, this adult underwent dynamic electronic examination of nose (Figures 1A, B) and MRI examination of sinus due to nasal bleeding. Examination results suggested sinusitis and a neoplasm appeared in the right nasal cavity, which was considered as a nasal polyp. Unfortunately, these early abnormal signals do not attract enough attention from doctors. Untypical clinical manifestations and insufficient understanding of ONB may be important reasons for the failure of timely diagnosis and treatment of this patient. Thus, when the symptoms of persistent nasal congestion and repeated nasal bleeding appear, clinicians should not only consider common diseases and frequentness, but also be alert to the possibility of malignant tumors. Considering the aggressive nature and high recurrence rate of ONB, timely surgery and adjuvant radiotherapy or chemotherapy may contribute to alleviate symptoms and improve survival (37).

In conclusion, this is the first report of PPN induced by ONB, which not only expanded the clinical characteristics of ONB but also highlighted the importance of early and comprehensive tumor screening for the diagnosis of PPN.

## Data availability statement

The raw data supporting the conclusions of this article will be made available by the authors, without undue reservation.

## Ethics statement

The studies involving human participants were reviewed and approved by Tongji Medical College, Huazhong University of Science and Technology (Approved No. of Ethic Committee: TJ-IRB20220617). The patients/participants provided their written informed consent to participate in this study. Written informed consent was obtained from the individual(s) for the publication of any potentially identifiable images or data included in this article.

## Author contributions

WL collected patient data. YW completed the article writing. ZZ and YF revised the manuscript. WS followed up the patients. DL and XZ searched the literature. PZ checked and revised the pathological description search section of article. All authors contributed to the article and approved the submitted version.
